# Non-parametric representation and prediction of single- and multi-shell diffusion-weighted MRI data using Gaussian processes

**DOI:** 10.1016/j.neuroimage.2015.07.067

**Published:** 2015-11-15

**Authors:** Jesper L.R. Andersson, Stamatios N. Sotiropoulos

**Affiliations:** FMRIB Centre, University of Oxford, UK

**Keywords:** Diffusion MRI, Gaussian process, Non-parametric representation, Multi-shell

## Abstract

Diffusion MRI offers great potential in studying the human brain microstructure and connectivity. However, diffusion images are marred by technical problems, such as image distortions and spurious signal loss. Correcting for these problems is non-trivial and relies on having a mechanism that predicts what to expect. In this paper we describe a novel way to represent and make predictions about diffusion MRI data. It is based on a Gaussian process on one or several spheres similar to the Geostatistical method of “Kriging”. We present a choice of covariance function that allows us to accurately predict the signal even from voxels with complex fibre patterns. For multi-shell data (multiple non-zero *b*-values) the covariance function extends across the shells which means that data from one shell is used when making predictions for another shell.

## Highlights

• We suggest using a Gaussian process (GP) to model diffusion MRI data.
• The GP is defined by a covariance function and a set of hyperparameters learned directly from the data.
• It can be used to make predictions that can subsequently be used in for example distortion correction.

## Introduction

Diffusion weighted MR imaging makes it possible to map the micro-structure and the connectivity of the living human brain. It proceeds by acquiring a set of echo planar images (EPI), each with the signal spoiled by a gradient such that the signal is lower in areas/voxels where water diffuses freely in the direction of that gradient. By acquiring many such images it is possible to build a profile of diffusivity in “any” direction for each voxel which can subsequently be used to probe the underlying microstructure and estimate white matter tracts by following the path of greatest diffusivity.

However, diffusion imaging is also marred by technical problems such as image distortions, subject movement and spurious signal loss caused by macroscopic movement during the diffusion encoding. Correcting for distortions and movement is a non-trivial problem since the distortions depend on the diffusion gradient and hence are different for each volume (see for example Andersson and Skare, 2002 or Rohde et al., 2004). Image registration based solutions are difficult since each volume will also have a different contrast and often (when applying strong diffusion weighting) poor SNR. The spurious signal loss will typically affect a whole, or a substantial part of a, slice but can be difficult to detect. It entails detecting a “smaller than expected” signal in a slice, but that hinges on knowing what to expect, which is non-trivial.

This paper describes a new way to model and predict diffusion signal from MR experiments. Unlike parametric models (Panagiotaki et al. (2012)) like for example the diffusion tensor (Basser et al. (1994)) or the ball-and-stick model (Behrens et al. (2003)), the model we propose will not yield biologically relevant parameters that are of value in their own right. Instead it is only used for making predictions about observed or unobserved measurements, which is something we will utilise in two related papers for:

• Correcting for distortions and subject movement by alignment of an observed volume to a predicted volume.
• Detection and replacement of outliers, typically signal loss caused by coherent movement during the diffusion encoding.

The representation we propose to use to describe the diffusion signal is a Gaussian process (GP). We should note that short preliminary descriptions of the distortion correction application of the Gaussian process predictor have been given in Andersson et al. (2012) and Sotiropoulos et al. (2013) and of its application to outlier detection in Andersson and Sotiropoulos (2014). The predictor forms the backbone of many of the preprocessing steps of the state-of-the-art data collected in the Human Connectome Project (Van Essen et al. (2013)). In this paper we describe in detail the underlying theory of the GP predictor and illustrate its working principles.

## Theory

### Gaussian processes

#### Introduction to Gaussian processes

Let us say we have a stochastic variable *Y* that is distributed as

(1) $$Y\sim N\left(\mu ,\sigma^{2}\right)$$

*i.e.* has a Gaussian distribution. This means that if we were to take an observation *y* from *Y*, we would expect to see a value “not too far off” *μ* and if we were to make a series of observations we would expect 68% of those to fall in the range [*μ* − *σ*, *μ* + *σ*].

A Gaussian process extends the concept of a stochastic variable to a stochastic function. Analogously to the stochastic variable above, we can say that a stochastic function *f*(*x*) is distributed as

(2) $$f\left(x\right)\sim GP\left(m\left(x\right),k\left(x,x^{\prime }\right)\right)$$

*i.e.* as a Gaussian process (Rasmussen and Williams (2006)) with a mean function *m*(*x*) and a covariance function *k*(*x*, *x*′). To understand Gaussian processes it can be useful to consider a *p*-dimensional stochastic variable distributed as

(3) $$Y\sim N_{p}\left(\mu ,\Sigma \right)$$

*i.e.* according to a multivariate normal (MVN) distribution characterised by a *p* × 1 mean vector ***μ*** and a *p* × *p* covariance matrix **Σ**. If we were to take a sample **y** from **Y** we would now expect **y** to be “close” to ***μ***, and if we were to take many samples we would expect most of them to fall within the confidence contours given by **Σ**. The role of **Σ** in this context is to describe how variable the different elements of **Y** are, *and* also how they covary. If for example **Σ**_12_ has a large positive value it is less surprising if both *y*_1_ and *y*_2_ have a value larger than *μ*_1_ and *μ*_2_ (or smaller) than if one of them is larger and the other smaller.

Analogously one could take a sample from *f*(*x*) and we would expect that sample to be “close” to the mean function *m*(*x*). And how surprising we would find deviations from *m*(*x*) depends on *k*(*x*, *x*′). In contrast to the MVN case this sample is a continuous function, *i.e.* it has a value for each *x*, though it may not have any parametric form. Another important distinction is that *k*(*x*, *x*′) is a continuous function with a value for any pair (*x*, *x*′) of *x*-values. Also, because *x* is a continuous variable it is meaningful to define a distance between two points *x* and *x*′, unlike the case of the elements of **y** where *y*_1_ and *y*_2_ could represent completely different entities.

Just as most applications of Gaussian distributions are inverse problems, where given a sample **y** one wants to find estimates for *μ* and *σ*^2^, most applications of Gaussian processes aim to estimate the mean function *m*(*x*) given some set of observed pairs (*x*_*i*_, *f*_*i*_) (often called the “training data” or the “training set”). The next section will explain how that is achieved.

#### Making predictions (estimating *m*(*x*))

For this section we will assume that there is a covariance function *k*(*x*, *x*′) that is known to us. How we actually find *k*(*x*, *x*′) will be the subject of the next section.

As stated above, *m*(*x*) may not have a parametric form, and even if it did it is not known to us. Given that, how can a continuous function on *x*, *i.e.* one where for any arbitrary value *x* we can calculate a value *f*(*x*), be meaningfully described? To answer that we first reorganise the training data into a vector of *n* observations of the independent variable which we call **x** and a vector of observations of the function (dependent variable) that we call **f**. We can then write the joint probability of the training data and any (unobserved) pair (x*, f*) as

(4) $$\left[\begin{array}{l} f\\ f^{*} \end{array}\right]\sim N_{n+1}\left(0,\left[\begin{array}{ll} K\left(x,x\right) & k\left(x,x^{*}\right)\\ k\left(x^{*},x\right) & k\left(x^{*},x^{*}\right) \end{array}\right]\right)$$

where **K**(**x**, **x**) is an *n* × *n* matrix of covariances between all the points in the training data, **k**(**x**, x*) is an *n* × 1 vector of covariances between the unobserved point x* and the training data and where *k*(x*, x*) is simply the variance at the point x*. It may seem counter intuitive that the MVN above has a zero mean, but that only means that *f*(*x*) is assumed to have a zero mean when averaged over all *x*. Typically one just subtracts the mean ($\bar{f}$) from **f** and then add it back to f*, which is analogous to what is often done in “traditional” regression.

In Eq. (4) everything is known except f* so in order to maximise the joint probability we just need to maximise the probability of f* conditional on x*, **x** and **f** which can be expressed as

(5) $$p\left(f^{*}|x^{*},x,f\right)=N(k\left(x^{*},x\right)K{\left(x,\;x\right)}^{-1}f,k\left(x^{*},x^{*}\right)-k\left(x^{*},x\right)K\left(x,x){}^{-1}k\left(x,x^{*}\right)\right)$$

and the value of f* that maximises it is of course the expectation **k**(x*, **x**)**K**(**x**, **x**)^− 1^**f**. This is similar to the E-step of the EM algorithm (Dempster et al., 1977) for MVN data with missing observations.

The estimation given by Eq. (5) assumes that the training data is “perfect”, *i.e.* that there is no uncertainty in the observations **f** of the dependent variable. The covariance function *k*(*x*, *x*′) does *not* model any error in the data, but the variability of the function itself. If we were to calculate **k**(x*, **x**)**K**(**x**, **x**)^− 1^**f** for “all” values of x* we would see that the resulting plot passed exactly through all of the training points (*x*_*i*_, *f*_*i*_).

Hence, in order to be able to use this with our data, which are always noisy, we need to complement it with a model for the measurement error. This means that Eq. (4) changes to

(6) $$\left[\begin{array}{l} f\\ f^{*} \end{array}\right]\sim N_{n+1}\left(0,\left[\begin{array}{ll} K\left(x,x\right)+\sigma^{2}I & k\left(x,x^{*}\right)\\ \;k\left(x^{*},x\right) & k\left(x^{*},x^{*}\right) \end{array}\right]\right)$$

and the conditional expectation and variance change to

(7) $$\hat{f}\left(x^{*}\right)=k\left(x^{*},x\right){\left(K\left(x,x\right)+\sigma^{2}I\right)}^{-1}f$$

and

(8) $$Cov\left(\hat{f}\left(x^{*}\right)\right)=k\left(x^{*},x^{*}\right)-k\left(x^{*},x\right){\left(K\left(x,x\right)+\sigma^{2}I\right)}^{-1}k\left(x,x^{*}\right)$$

respectively, where *σ*^2^ is the variance of the observation error (it will be explained later how we estimate *σ*^2^) and where **I** is the *n* × *n* identity matrix.

Eq. (7) provides a way to make predictions for both observed and unobserved points x* given some observations *x* and *f*, *provided* that we know the function *k*(*x*, *x*′).

#### Finding *k*(*x*, *x*′)

There are a lot of suggestions for covariance functions *k*(*x*, *x*′) in the literature about Gaussian processes. They are typically devised so that nearby points have a larger positive covariance than points further apart thereby imposing smoothness on the function. For a function *k*(*x*, *x*′) to work as a covariance function it needs to produce a positive definite matrix **K**(**x**, **x**) for any set **x** of points in the domain of *f*(*x*) (see *e.g.* Genton, 2001).

Many existing covariance functions, such as for example the squared-exponential (Rasmussen and Williams, 2006), are parametric, *i.e.* they have a number of free parameters (often referred to as hyperparameters) whose values determine the detailed properties of the resulting GP. The task of finding a suitable covariance function for one's application/data entails not only choosing the “right” parametric form but also, possibly on a per data set basis, suitable values for the parameters. This can be achieved by marginal likelihood maximisation (Rasmussen and Williams, 2006), or by leave-one-out methods (Sundararajan and Sathiya Keerthi, 2001).

### Gaussian processes for diffusion data

This section builds the case for the covariance functions we suggest for diffusion data.

**Fig. 1:**
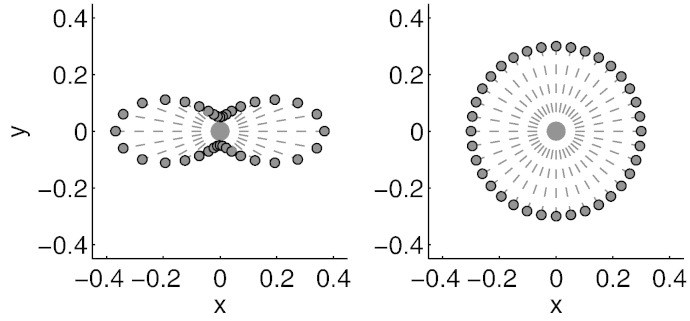
Simulated (2D) examples of diffusion weighted measurements. The direction along which diffusion weighting was applied is shown by a dashed line and the measured signal along that direction is indicated by the distance of the round marker from the origin. The left panel shows the case where diffusivity is three times greater along the *y*-axis than along the *x*-axis. The right panel demonstrates the case where diffusivity is equal in all directions. The extension of this to 3D is straightforward, though a little tricky to demonstrate in a figure. If we extend the figure in the left panel to 3D and assume that the diffusivity along the direction perpendicular to the paper is the same as for the *x*-direction, the points sampled on the resulting surface would form a “red blood cell” seen from the side. Correspondingly in the right panel if we assume equal diffusivity in all three directions the resulting surface would be a sphere.

Diffusion data is acquired by, for each voxel, observing the signal after applying a diffusion weighting along a specific direction. Hence the data can be seen as a response variable (the signal) acquired on the surface of a sphere. The weighting is typically characterised by a *b*-value that specifies the strength of diffusion weighting and a unit length vector **g** that specifies the direction. The signal is affected by the local diffusion of water molecules such that a high diffusivity along **g** leads to a small signal. A full diffusion protocol consists of multiple measurements along different directions aimed at characterising the diffusion along “any” direction. A two-dimensional demonstration of how the diffusion signal might look can be seen in Fig. 1. This shows two important aspects of the diffusion signal:

• The signal changes smoothly as the angle of the diffusion weighting direction changes.
• The signal is axially symmetric, *i.e.* the signal along **g** is identical to the signal along − **g**.

Because the diffusion signal lives on a sphere, it is a good match for techniques that have been developed and used for geostatistics and meteorology where a special case of GPs observed on a sphere is known as “Kriging” (Wackernagel, 2003). For these techniques the covariance is often defined as a function of an angle *θ* between two vectors from the centre of the sphere to **x** and **x**′. These vectors are easily recognised as the **g**-vectors described above. Two popular covariance functions in geostatistics are the “Exponential model”

(9) $$C\left(\theta \right)=e^{-\theta /a}\;for\;0\leq \theta \leq \pi$$

where *a* is a positive scale parameter, and the “Spherical model” where *a* is again a positive scale parameter that here determines the “distance” at which *θ* the covariance goes to zero:

(10) $$C\left(\theta \right)=\left\{\begin{array}{ll} 1-\frac{3\theta }{2a}+\frac{\theta^{3}}{2a^{3}} & \mathrm{if}\;\theta \leq a\\ 0 & \mathrm{if}\;\theta >a \end{array}\right)\text{.}$$

Both of these are “valid” covariance functions (Huang et al., 2011) on the sphere, *i.e.* they will yield invertible matrices **K** and the marginal likelihood will exist for any data. For diffusion data we need to modify the definition of *θ* since we want the model to be symmetrical on the sphere. We do this by defining *θ* for two unity length diffusion gradient vectors **g** and **g**′ as

(11) $$\theta \left(g,g^{\prime }\right)=\arccos\left|\langle g,g^{\prime }\rangle \right|\text{.}$$

This is equivalent to extending both vectors also in the negative direction and choosing the smallest of the two angles between the resulting crossing lines.

#### Single shell data

**Fig. 2:**
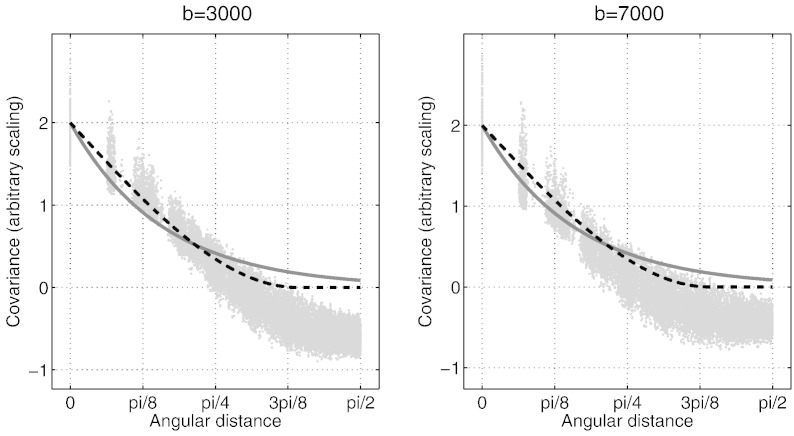
The empirically observed covariance *versus* angle between diffusion weighting directions (*b*-vectors) for the HCP *b* = 3000 (left) and *b* = 7000 data sets described in Table 1. Each point represents one pair of *b*-vectors and the covariance is calculated across all intra-cerebral voxels. The points with zero angle corresponds to the variance (pooled across all voxels) for each direction. The solid grey line corresponds to the exponential (Eq. (9)) and the dashed black line to the spherical (Eq. (10)) covariance function. These are “Chi-by-eye” lines and are there to demonstrate their respective general appearance in relation to the empirically observed covariance. The same “length scale” parameters were used for both plots (*a* = 1.23 and *a* = 0.5 for the spherical and the exponential functions respectively).

When all the diffusion weighted measurements are performed with the same *b*-value, data are said to be collected on a single shell, which is then very similar to the geostatistical application of Kriging. One can obtain an idea about the form of the covariance function by calculating a sample covariance and plotting the elements of that matrix against *θ*. The resulting plot can be seen in Fig. 2, and both the exponential and the spherical covariance functions capture the general appearance of the observed covariance, with the spherical model possibly looking a little better. To estimate the optimal hyperparameters for each model one can use marginal likelihood maximisation (also known as type II maximum likelihood) (Rasmussen and Williams, 2006) which maximises

(12) $$\log p\left(y|\beta ,M\right)=-\frac{1}{2}y^{T}K_{y}^{-1}y-\frac{1}{2}\log\left|K_{y}\right|+c$$

where **y** is the signal from one voxel for all the diffusion directions, where *β* = [*λ a σ*^2^] and where **K**_*y*_ = **K** + *σ*^2^**I** and **K** is the matrix with elements *K*_*ij*_ = *λC*(*θ*_*ij*_; *a*) where *C*(*θ*; *a*) is given by Eq. (9), (10) depending on which model M is being considered. In these models, *a* is a “distance scale” parameter determining how fast the covariance decreases as one moves along the surface of the sphere, *λ* is a “signal scale” parameter which determines the variability of the signal and *σ*^2^ determines the uncertainty of the measured values **y**. When estimating the hyperparameters the model was reparameterised so that $\tilde{\lambda }=e^{\lambda }$ and $\tilde{\sigma^{2}}=e^{\sigma^{2}}$ were estimated rather than *λ* and *σ*^2^ themselves so as to avoid the possibility of negative scaling or variance.

The term “marginal likelihood” seems a little counter intuitive since it is not immediately clear what is being marginalised over. “Normally” when estimating the values of some hyperparameters the marginalisation occurs over the lower level parameters of the model. In the case of Gaussian processes the “parameters” are all possible functions *f*(*x*) and we recommend chapters 2 and 5 of Rasmussen and Williams (2006) for an explanation of this.

When maximising Eq. (12) one finds the optimal hyperparameters *β* for the particular voxel from which **y** is taken. However one would like to find a single *β* for all voxels. To find that, Eq. (12) is summed over all (or at least a sizeable subset of all) voxels, which is equivalent to multiplying the likelihoods over voxels (Minka and Picard, 1999).

Eq. (12) still cannot be used to choose between the models. For that we would need the model evidence

(13) $$p\left(M_{i}|y\right)\propto p\left(y|M_{i}\right)=\int_{\beta }p\left(y|\beta ,M_{i}\right)p\left(\beta |M_{i}\right)d\beta$$

where *p*(*β*|M_*i*_) is the prior distribution of *β*. In order to calculate the integral in Eq. (13) we use Laplace's approximation which entails finding the *β* which maximises *p*(**y**|*β*, M_*i*_), calculating the Hessian at that point *β*_0_ and approximating *p*(*β*|M_*i*_) by a Gaussian distribution centred on *β*_0_ and the covariance given by the inverse Hessian. We leave the details of those calculations to Appendix A, B.

It is true that Laplace's equation is an approximation, but it should be noted that the ability of a Gaussian process to make useful predictions (*i.e.* to estimate the mean function through Eq. (7)) is not strongly dependent on the exact form of the covariance function (Press et al., 2007).

#### Leave-one-out methods

In addition to the marginal likelihood maximisation we also implemented and tested methods based on maximising the ability to predict unobserved data. In the interest of space we will not present any results derived with those methods and simply mention that we implemented and tested the methods referred to as Cross-Validation (CV), Geisser's Surrogate Predictive Probability (GPP) and Geisser's Predictive mean Square Error (GPE) as described in Sundararajan and Sathiya Keerthi (2001). We found that both CV and GPP yielded hyperparameters that obtained good predictions and hence they are both part of our implementation. In contrast to Sundararajan and Sathiya Keerthi (2001) we found that GPE did not perform well.

#### Multi-shell data

Increasingly diffusion data is acquired with two or more different non-zero *b*-values (see for example Alexander et al., 2006; Aganj et al., 2010; Sotiropoulos et al., 2013 or Setsompop et al., 2013). This type of data is referred to as “multi-shell” data. The logic behind this name is that the data collected for each *b*-value forms a closed 2D surface embedded in 3D space where the surface resulting from the high *b*-value is completely enclosed inside the surface formed by the low *b*-value. The rational behind such an acquisition is that high *b*-values give more angular contrast and higher “diffusion resolution” but lower SNR than low *b*-values.

A general strategy for defining covariance functions is to construct new functions from products or linear combinations of existing ones (Rasmussen and Williams, 2006). For multi-shell data two points may differ on two axes *θ* and Δ*b* where *θ* is defined as above and Δ*b* is the difference in *b*-value between the two points. A “natural” covariance function for multi-shell data would hence be

(14) $$k\left(x,x^{\prime }\right)=C_{\theta }\left(\theta \left(g,g^{\prime }\right);a\right)C_{b}\left(\left|b-b^{\prime }\right|;\ell \right)$$

where *C*_*θ*_ is the covariance function we defined above for the single shell case, where *C*_*b*_ is some candidate smooth function describing how the covariance changes along the *b* direction and where *ℓ* is some set of hyperparameters for *C*_*b*_.

We have chosen the squared-exponential (Rasmussen and Williams, 2006) covariance function for *C*_*b*_ and have used the log of the *b*-values as the measure of distance along the *b*-direction. Hence, *C*_*b*_ is given by

(15) $$C_{b}\left(b,b^{\prime };\ell \right)=\exp\left(-\frac{{\left(\log b-\log b^{\prime }\right)}^{2}}{2\ell^{2}}\right)\text{.}$$

Multi-shell acquisition schemes typically consists of a small finite set (often 2–3) of shells in the *b* direction. For each of those shells we allow for a unique measurement error. If we assume that we have two shells, the full **K**-matrix can be written as

(16) $$K=\left[\begin{array}{cc} \lambda C_{\theta }\left(\theta \left(G_{1}\right);a\right)+\sigma_{1}^{2}I & \lambda C_{\theta }\left(\theta \left(G_{2},G_{1}\right);a\right)C_{b}\left(b_{2},b_{1};\ell \right)\\ \lambda C_{\theta }\left(\theta \left(G_{1},G_{2}\right);a\right)C_{b}\left(b_{1},b_{2};\ell \right) & \lambda C_{\theta }\left(\theta \left(G_{2}\right);a\right)+\sigma_{2}^{2}I \end{array}\right]$$

where the hyperparameters that need to be estimated are *β* = [*λ a ℓ σ*_1_^2^ *σ*_2_^2^], where *θ*(**G**, **G**′) is a matrix-valued function with all the angles between the **g**-vectors in the sets **G** and **G**′, where *C*_*θ*_ is given by Eq. (9), (10) and where *C*_*b*_ is given by Eq. (15). Eq. (16) is trivially extended to the *N*-shell case and the number of hyperparameters goes as 3 + *N*.

The same marginal likelihood maximisation that was described for the single shell case can be used to determine the hyperparameters of the multi shell case, as can either of the prediction based methods referred to in Section 2.2.2.

**Fig. 3:**
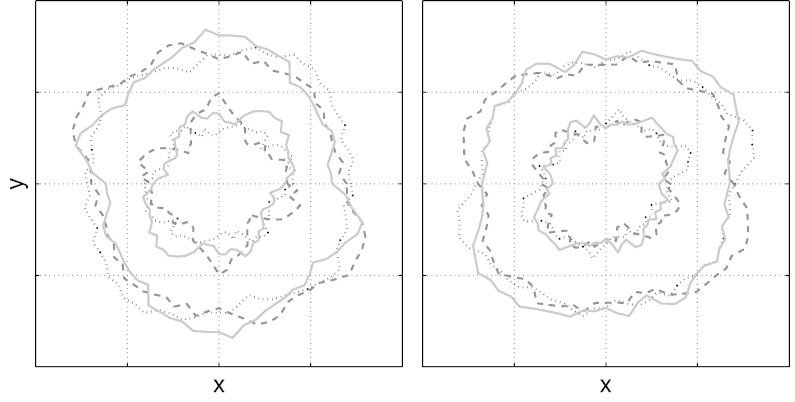
Examples of prior shapes (cut at arbitrary plane) generated using hyperparameters estimated from the HCP b = 1500 data (outer shell) and the HCP b = 5000 (inner shell). The solid, dashed and dotted shapes represent three different realisations drawn from the distribution of possible shapes. On the left hand side the priors were drawn independently for the two shells and on the right they were drawn from the multi-shell model (Eq. (16)). Note how in the absence of data, the expected shape is approximately spherical (isotropic diffusion) and that the (relative) variability is greater for the inner shell. Note also that for the multi-shell model (right hand size) the shapes covary across the shells.

In Fig. 3 we demonstrate the “Prior shapes” given by the single- (left panel) and multi-shell (right panel) models. A prior shape is a shape that has been drawn from the distribution of plausible shapes given the form of the covariance function and the values of the hyperparameters. Any shape has a probability of being drawn that is proportional to its prior probability (*i.e.* in the absence of any data). It can be seen that the prior favours approximately spherical shapes in the absence of any evidence to the contrary.

#### A note on optimisation

It is suggested, for example in Rasmussen and Williams (2006), that an optimisation method that uses derivative information should be used when finding the hyperparameters that maximise Eq. (12). The reason for that is that such methods typically use fewer steps, and when the cost of calculating the derivatives is small/moderate compared to calculating the functions itself (as is the case for Eq. (12)) then execution time can be much shorter. However, we found that for the multi-shell case a heuristic optimisation method such as the Nelder–Mead simplex method (Nelder and Mead, 1965) was frequently better at avoiding local maxima. Hence, that was the method we used for all optimisations in the present paper.

## Materials and methods

### 3.1 Diffusion data

In order to ensure that results are not specific to a particular scanner and/or protocol, data was taken from several studies performed by several groups. The common feature of all data sets is that they are at the upper end of what is usually acquired in terms of number of directions and *b*-values. Relevant key parameters are summarised in Table 1.

After acquisition, data was corrected for susceptibility induced distortions (in the case where data was acquired with reversed phase-encode directions) and eddy current induced distortions and subject movement. It may seem circular that we used our Gaussian process based method for distortions and movement to correct the data prior to using it in the present paper. How this is performed is briefly explained in Appendix C.

### Analysis

#### Single shell model selection

For each of the data sets three slices through the centre of the brain were selected and all the intra cerebral voxels of those slices were used. The hyperparameters for both models (given by Eqs. (9), (10)) were estimated by maximising the log marginal likelihood (Eq. (12)) summed over all voxels. The evidence was calculated for each model as described in Appendix A, B and for each data set, the Bayes factor comparing the two models was calculated.

#### Predictions

The model selected based on the previous section was used to make predictions of diffusion weighted volumes. Predictions were made both including the observed data for the predicted volume (in which case the GP performs a smoothing on the sphere) and excluding it (in which case the GP corresponds to an interpolation) in the training data.

In order to see how the model allows us to improve predictions for one shell by utilising information from other shells we subsampled one of the shells and calculated predictions for that shell in isolation (Eq. (9), (10)) and in the context of one or more other shells (Eq. (16)).

## Results

### Single shell model selection

All data sets showed a very strong preference for the spherical model (Eq. (10)) over the exponential model (Eq. (9)). Even for single voxels, the Bayes factor (Kass and Raftery (1995)) ranged from 1 (for voxels in CSF) to > 10,000 (white matter voxels with strongest preference). When estimating a single set of hyperparameters from a large selection (> 10,000) of intracerebral voxels, the resulting Bayes factor was so large that it approached the numerical precision of a double. This finding was independent of the data set used. It was therefore decided to use the spherical model for all further analysis.

**Fig. 4:**
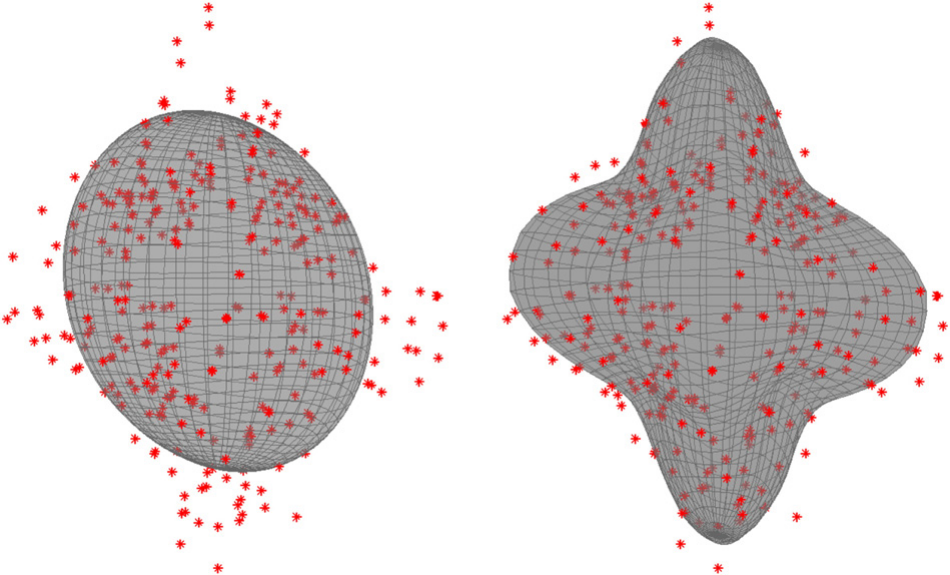
Example of predictions from a tensor fit (left) panel and from a Gaussian process fit (right) panel to a crossing fibre voxel in the Centrum Semiovale in the region where the superior longitudinal fasciculus II crosses the corticospinal tract. The data is a single shell with a *b*-value of 3000. The data is shown as red dots and the model prediction as a grey surface. As expected the Gaussian process shows a much better ability to predict the data from such voxels despite being even faster to calculate than the tensor prediction.

Fig. 4 shows the predictions made by the tensor model and the suggested GP. The data in Fig. 4 comes from a white matter voxel with a three-way crossing (each local minimum on the model fit in the right panel represents a fibre direction). It can be seen that the Gaussian process is able to model the structure of the signal very well without any obvious “overfitting”.

**Fig. 5:**
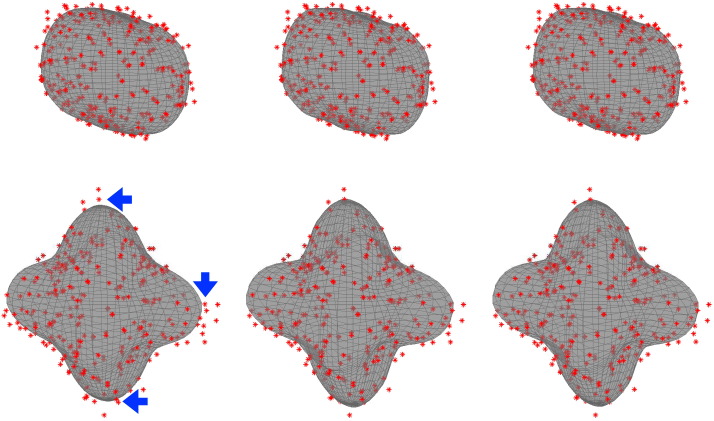
The top row shows the signal (with a *b*-value of 3000) from a cortical grey matter voxel and the bottom row from a white matter voxel with a three-way crossing. The left most column shows data and predictions obtained when deriving the hyperparameters from the grey matter voxel, the middle column when deriving them from the white matter voxel and the right most column when deriving them jointly from both voxels. It can be seen (lower left sub-figure) that the modelling of the white matter voxel is affected somewhat negatively when using the hyperparameters estimated from the grey matter voxel. The arrows point to data points where it can be seen that the distance to the model fit is greater than for the other two columns. When using the jointly derived hyperparameters, the GP (lower right sub-figure) manages to capture the features of the data equally well as when the white matter derived hyperparameters are used (lower middle sub-figure).

In Fig. 5 we demonstrate the impact of the hyperparameters on the ability of the GP to model the data. It shows data from two voxels, one in grey matter and one from a white matter region with complex architecture (*i.e.* crossing fibres). Three sets of hyperparameters are estimated, from the grey matter voxel, from the white matter voxel and jointly from both. All three sets of hyperparameters are subsequently used to model data from both voxels. It can be seen that both the hyperparameters estimated from the white matter and those estimated from both yields processes that are able to adequately model either voxel.

**Fig. 6:**
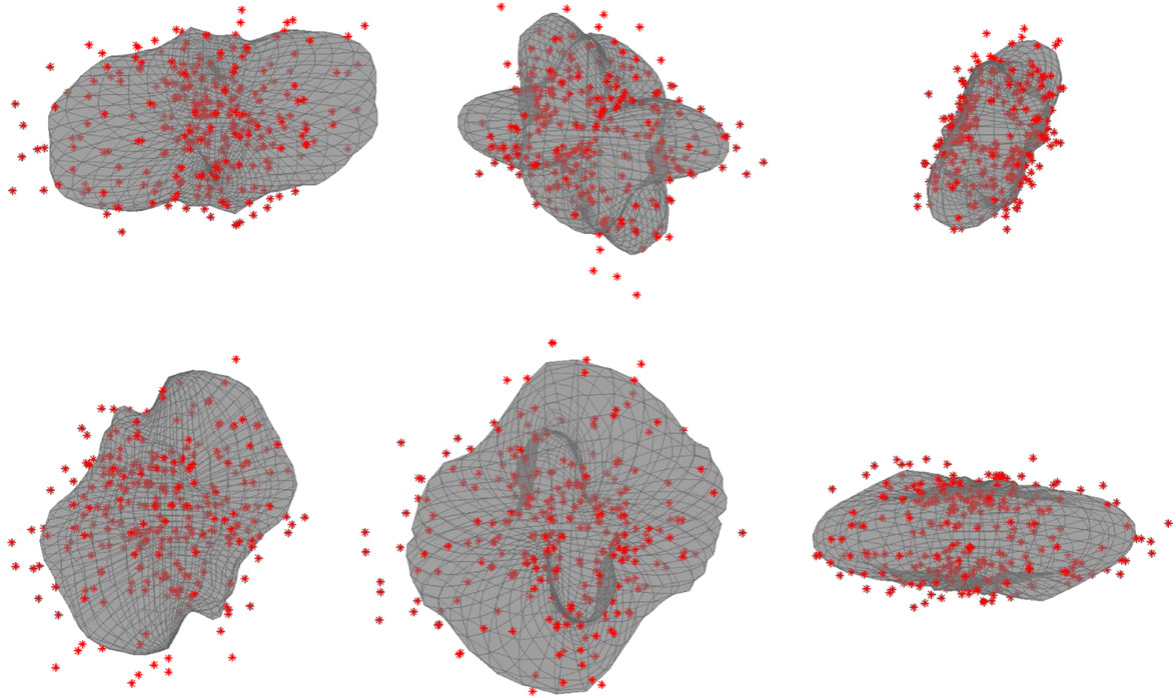
The figure shows data (red) and Gaussian process (GP) fit (grey) from a b = 7000 shell from the pilot phase of the HCP. The six panels correspond to six randomly selected voxels in a transversal slice at the level of the Centrum Semiovale. The hyperparameters for the GP was the same for all voxels (and calculated from a random selection of 1000 intracerebral voxels). It can be seen that the GP has been able to successfully model the signal from the six voxels despite exhibiting vastly different signal profiles. It can for example be appreciated from the signal that the top panel in the middle column corresponds to a three-way crossing fibre, the top panel in the right column a two-way crossing fibre and the lower panel in the middle column a single (dominating) fibre. The top-left and bottom-right panels correspond to grey matter voxels.

The Gaussian process is able to model high *b*-value data from voxels with vastly different signal profiles. To show that, we estimated hyperparameters from a random selection of 1000 intracerebral voxels in a data set acquired with a *b*-value of 7000. The resulting Gaussian process was used to model data from six randomly selected voxels from a plane at the level of the crossing of the superior longitudinal fasciculus II and the cortico spinal tract. The results are shown in Fig. 6.

### Multi-shell model

**Fig. 7:**
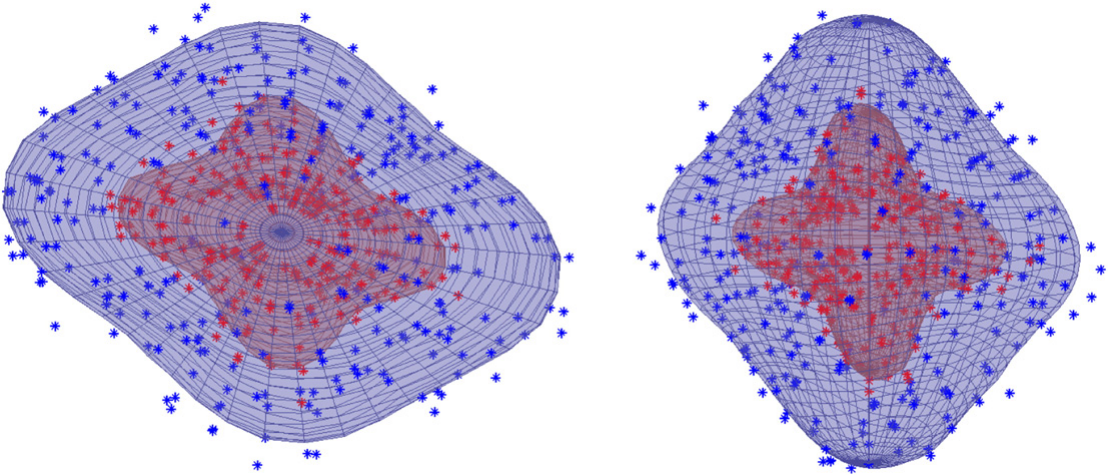
Example of multi-shell predictions. The same voxel as in Fig. 4 but using two other shells with *b*-values of 1500 (blue) and 5000 (red). Both panels show the same data rotated to demonstrate it more fully. In the multi-shell model the data from one shell will impact on the predictions about the other shell and yet it is clear from this figure that the predictions for the high *b*-value shell has additional detail and is not just a scaled version of the lower *b*-value predictions.

Fig. 7 shows data and the resulting predictions for two shells with *b*-values 1500 and 5000 when using model (16). The prediction at any point (any point on either of the surfaces) is a linear combination of the data points with higher weights given to the points with similar *θ* and *ϕ* (in spherical coordinates) and higher weights to points on the same shell.

**Fig. 8:**
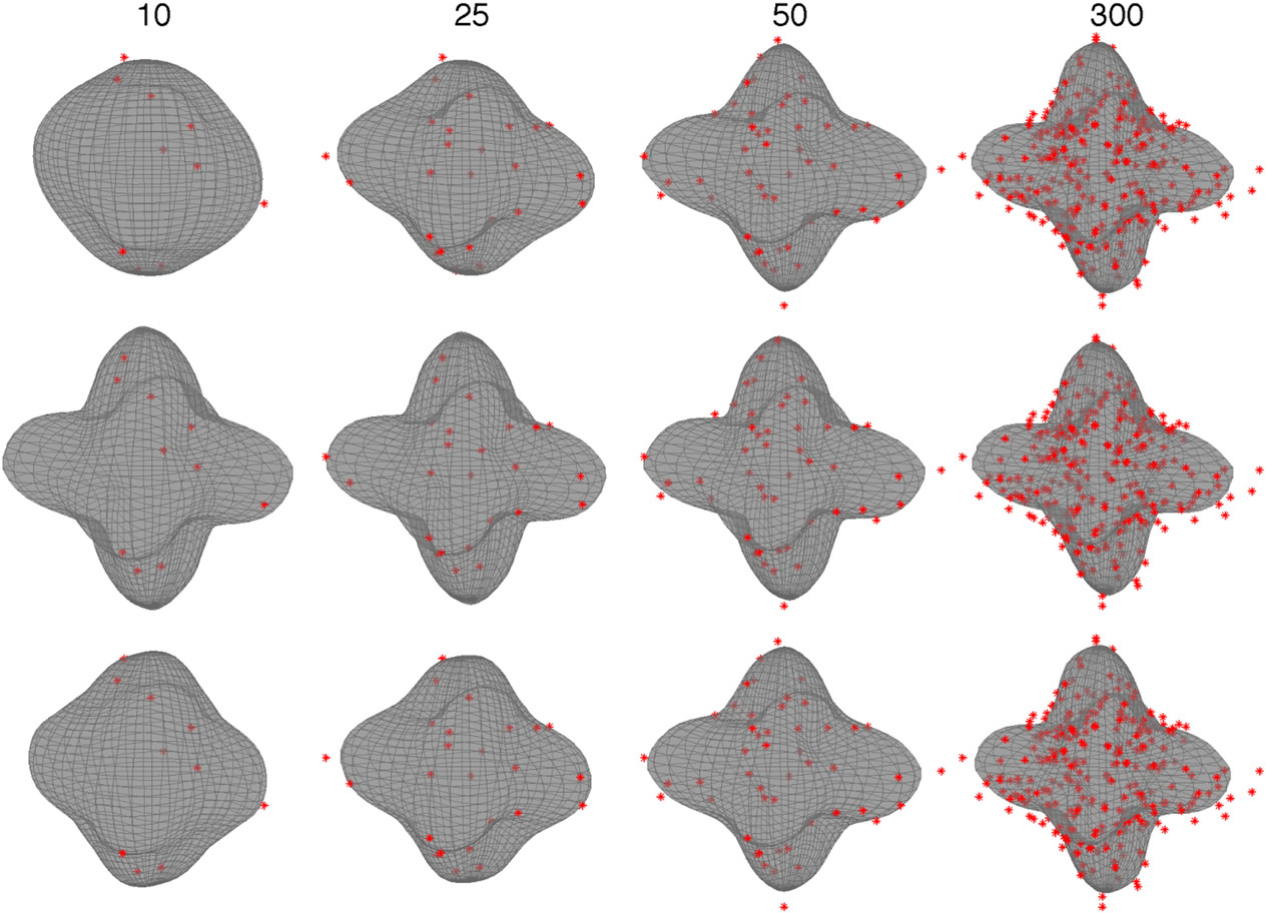
This figure demonstrates the predictions for a *b* = 5000 voxel when considering only the *b* = 5000 data points (top row) or when using also the *b* = 3000 data (middle row) or the *b* = 1500 data (bottom row). The predictions are shown when using only the first 10, 25 and 50 points from a set of 300 as well as when using all 300 points. It can be seen (middle row) that the ability to make meaningful predictions from a paucity of data is very much improved when utilising information from the neighbouring shell (*b* = 3000). It can also be seen (bottom row) that when the “supporting shell” is further away, its impact is smaller, but still appreciable when the number of data points is 25 or less.

The ability to improve the predictions for one shell by utilising information from (an)other shell(s) is demonstrated in Fig. 8.

### Predictions

**Fig. 9:**
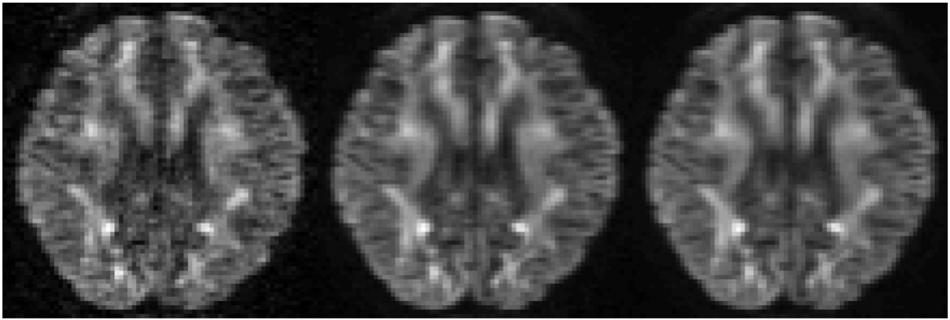
Examples of observed and predicted images for the single shell model. The left panel shows an image acquired with a *b*-value of 3000 and the diffusion gradient [1 0 0], the middle panel shows the Gaussian process prediction when the observed image was part of the training data (smoothing) and the right panel when the observed image was not (interpolation).

Fig. 9 demonstrates that the model was capable of making very accurate predictions both when including and excluding the observed data corresponding to the prediction.

## Discussion

We have demonstrated the use of Gaussian processes for modelling and making predictions about diffusion data. For each new data set a small number (three for most acquisition protocols) of hyperparameters have to be non-linearly estimated and following that all voxels can be modelled using a fast linear method. Despite its speed and simplicity it can model voxels with several crossing fibres.

Gaussian processes are sometimes touted as “model free”, and therefore as a solution to the problem where one has some data that one wants to model but one doesn't have a good theoretical argument for choosing one model over another. This is partially true, but when using Gaussian processes the task has shifted from finding a parametric model for the function to finding a parametric model for the covariance function *k*(*x*, *x*′).

It may seem counterintuitive that a Gaussian process with a single set of hyperparameters (estimated from voxels that represent a mixture of tissue types) can model highly structured data from crossing fibre white matter (such as shown in Fig. 4, Fig. 5, Fig. 6, Fig. 7, Fig. 8) as well as from grey matter or CSF. To understand that, it should be realised that the covariance function acts only as a prior on the shapes that can be modelled by the GP. The estimated hyperparameters will be dominated by the white matter signal, because it is only for white matter that there is appreciable signal variation on the sphere. Even so, the most likely shape before observing any data (prior shape) will be spherical (this is true for all “proper” covariance functions on the sphere) so there will be no problems modelling the grey matter or CSF. At the same time there will be enough signal variability (parametrised by *λ*) to adequately capture the structure in white matter.

The reason we have opted for a Gaussian process rather than some previously published parameteric model (see Panagiotaki et al. (2012) for examples of biophysical models) is more pragmatic than for its presumed “model freeness”. A Gaussian process is linear, like the single diffusion tensor model after log-transformation of the data, which means it is practical (*i.e.* fast) to incorporate into a framework where the model has to be re-estimated several times as part of an iterative procedure. In fact, even for the HCP data where each prediction is an inner product of two 300 × 1 vectors, it is twice as fast to calculate as the tensor-based prediction, and for data sets with less points, the difference becomes greater. At the same time it is not so strictly limited in terms of what it can model as the single tensor model, which means that it can better model the signal in areas with for example crossing fibres. It is also less inherently sensitive to artefactual signal loss than the log-transformed least-squares tensor model.

There are other methods for modelling the diffusion signal that are less restrictive than the diffusion tensor and still computationally feasible, such as for example spherical harmonics (Descoteaux et al., 2006) or Watson direction functions (Rathi et al., 2009). Compared to these our approach offers the advantage of not having to decide on an order of harmonics or number of direction functions. It also offers (in common with Descoteaux et al., 2011) the ability to simultaneously model multiple shells with the estimates from one shell informing the others. Furthermore we aim to develop correction techniques that are as independent as possible of how the subsequent processing/analysis of the data is performed so as to avoid circularities. Hence we wanted to avoid commonly used models such as those mentioned above (Descoteaux et al., 2006 or Rathi et al., 2009). Apart from the circularity argument it is likely that for example a spherical/solid harmonics model with priors on the parameters to ensure spherical prior shapes could equally work for our purposes.

The way in which the Gaussian process can use information from one shell to aid in the prediction of another shell is through the observed covariances between the shells (which will determine the value of the *ℓ* hyperparameter in Eq. (15)). That means that if two shells are far apart, and especially if one of the shells has “low” *b*-values, the observed covariance will be small and the predictive power of one shell on the other will be small. This can be seen in Fig. 8 where it is demonstrated that a *b* = 3000 shell will have a substantial impact on the predictions made for a *b* = 5000 shell when there is a paucity of data, whereas a *b* = 1500 shell will have a much smaller, albeit non-zero, impact. It can also be seen that when the *b* = 5000 shell gets more data the impact of the other shells diminishes as the within-shell covariances starts to dominate.

As outlined in the Introduction section we plan to use this model for two purposes, the first being correction for eddy current distortions and subject movement. In general it is a difficult and largely unsolved problem how to register images acquired with different diffusion gradients because of their different information contents. At the same time it is often desirable to register them, because long study durations make it likely that the subject will have moved between some of the volumes, and because each volume tend to be distorted in a unique way that is determined by the direction and strength of the diffusion gradients (see for example Andersson and Skare (2011) for an overview). Our intended use of the Gaussian process model is to register the observed images to their predictions. Since the predictions are based on all (or a majority if *a* < *π*/2) volumes, the resulting prediction will be closer to the average space of the study both in terms of distortions and subject position, than the corresponding observed image. Hence, by iteratively nudging each volume closer to the corresponding prediction we obtain a registered set of images.

The second planned use for this model is outlier detection and replacement (briefly described in Andersson and Sotiropoulos, 2014). It is not uncommon for diffusion weighted images to suffer from a loss of signal, that may be quite severe. This is caused by (tiny) subject movement or pulsatile movement of the brain (Pierpaoli, 2011) during the diffusion weighting which causes a translation of the signal in *k*-space, potentially to partially outside the *k*-space window that is sampled. If uncorrected this signal loss will be interpreted as high diffusivity in the direction of the gradient of the affected volume and will bias tractography. There are methods for detecting such outliers (for example RESTORE, Chang et al., 2005) but these are all based on one specific model (typically the tensor) for the diffusion signal. Consider for example the left panel in Fig. 4 where the lack of model fit would imply a large number of outliers, but where the real issue is an inadequate model. The suggested Gaussian process in contrast is mainly data driven and independent of the particular model that is used for the subsequent analysis/tractography. It should be noted that more flexible models have been suggested for outlier detection (see for example Pannek et al., 2012) and that they would not suffer from the problems that the tensor model does.

## Conclusion

We have suggested a method for modelling the diffusion signal that enables us to make accurate predictions. It is based on a Gaussian process, is highly data driven and allows for multi-shell modelling.

## Figures and Tables

**Table 1:** The table shows a few key parameters for the data that was used for testing the GP. RP stands for Reversed Polarity and implies that each diffusion gradient was acquired twice with opposing phase-encode directions.

Scanner	*b*-Value	# of directions	Resolution (mm)	RP	Reference
Siemens Verio	1500	120	2^3^ mm^3^	Yes	
Siemens Trio	2500	124	2.2^3^ mm^3^	No	
1500
3000
Siemens Skyra	5000	300	2^3^ mm^3^	Yes	Uğurbil et al. (2013)
7000
MGH-HCP	10,000	198	1.5^3^ mm^3^	No	Setsompop et al. (2013)

## Appendix A

### A Laplace's approximation of model evidence

Given two models $M_{1}$ and $M_{2}$ that we wish to compare we need to calculate the model evidence for both models and choose the one with the greater evidence. A Gaussian process model is strictly speaking a two level model with parameters (being all possible functions *f*(*x*) as described in the main text) and hyperparameters *β*. For brevity the first level will be omitted here. The posterior over the hyperparameters *β* is given by

(A1) $$p\left(\beta |y,M\right)=\frac{p\left(y|\beta ,M\right)p\left(\beta |M\right)}{p\left(y|M\right)}\text{.}$$

Assuming uninformative priors *p*(*β*|M) a maximum a posteriori (MAP) estimate *β*_*mp*_ can be obtained by maximising *p*(**y**|*β*,M), *i.e.* Eq. (12) in the main text. One can then calculate the Hessian **H**_*mp*_, the matrix of second derivatives of Eq. (12), at the MAP point using Eq. (B2). The inverse of **H**_*mp*_ is a measure of the uncertainty of the estimate *β*_*mp*_ and under the (Laplace) assumption of a normal distributed posterior one can write

(A2) $$p\left(\beta |y,M\right)\approx N\left(\beta_{mp},H_{mp}^{-1}\right)\text{.}$$

The next step is to use this to approximate the model evidence. The model evidence for model *i* is

(A3) $$p\left(M_{i}|y\right)=\frac{p\left(y|M_{i}\right)p\left(M_{i}\right)}{p\left(y\right)}\text{.}$$

For the purpose of comparing different models *p*(**y**) is an uninteresting scaling factor and if one further assumes that there is no prior preference for one model over another the evidence becomes

(A4) $$p\left(M_{i}|y\right)\propto p\left(y|M_{i}\right)=\int p\left(y|\beta ,M_{i}\right)p\left(\beta |M_{i}\right)d\beta$$

but from Eq. (A1) it is known that

(A5) $$p\left(y|\beta ,M_{i}\right)p\left(\beta |M_{i}\right)\propto p\left(\beta |y,M\right)$$

which means that the integrand is, approximately, a normal distribution with its “width” depending on **H**_*mp*_^− 1^ and its peak height on *p*(**y**|*β*_*mp*_,M_*i*_)*p*(*β*_*mp*_|M_*i*_). Results for integrating a Gaussian yields

(A6) $$p\left(y|M_{i}\right)\approx \underset{\mathrm{Likelihood}}{\underset{︸}{p\left(y|\beta_{mp},M_{i}\right)}}\times \underset{\mathrm{Occam}\;\mathrm{factor}}{\underset{︸}{p\left(\beta_{mp}|M_{i}\right){\left(2\pi \right)}^{d/2}{\left|H_{mp}^{-1}\right|}^{1/2}}}$$

where *d* is the number of hyperparameters (length of *β*) for model $M_{i}$ and where it is implicit that **H**_*mp*_ pertains to the particular model in question.

The comparison between models $M_{i}$ and $M_{j}$ finally is performed using the Bayes factor

(A7) $$B_{ij}=\frac{p\left(y|M_{i}\right)}{p\left(y|M_{j}\right)}\text{.}$$

One critical aspect of the Bayes factor is *p*(*β*_*mp*_|$M_{i}$), *i.e.* that one needs prior distributions on the parameters for the different models and that the outcome of the model comparison can depend crucially on ones particular choices of priors. In the present paper uninformative priors were used in order to minimise their impact on the model comparison. For the parameter *a* a uniform prior on the interval [0, *π*] was used for all models. The parameters *σ*_*m*_^2^, *σ*_*n*(*i*)_^2^ and *B*_*ij*_ all represent entities related to variance and would scale as *α*^2^ if the data was rescaled by a factor *α*. The same rescaling would alter |**H**_*mp*_^− 1^|^1/2^ by a factor of *α* for each variance related parameter in the model. Therefore, in order to render the model comparison independent of rescaling of the data, 1/*σ* (and conversely $1/\sqrt{B_{ij}}$) was used. For the reparametrisation $\tilde{\beta }=e^{\beta }$ this corresponds to an (improper) prior $U\left(-\infty ,\infty \right)$ on $\tilde{\beta }$.

To make this concrete: For the spherical model the hyperparameters are [*σ*_*m*_^2^ *a σ*_*n*_^2^] and for a particular data set *β*_*mp*_ was [119 1.15 186]. That would make

(A8) $$p\left(\beta_{mp}|M_{i}\right)=\frac{1}{\sqrt{119}}\frac{1}{\pi }\frac{1}{\sqrt{186}}=2.14\times 10^{-3}\text{.}$$

For a more in-depth treatment of this we recommend MacKay (2003) and Kass and Raftery (1995).

### B Calculating the Hessian

The Hessian we need for the approximation of the model evidence is the matrix of second derivatives of the negation of log *p*(**y**|*β*) (where we have omitted the explicit dependence on model M) as defined by Eq. (12). The first derivatives of Eq. (12) are given in Rasmussen and Williams (2006) and are

(B1) $$\frac{\partial }{\partial \beta_{i}}\log p\left(y|\beta \right)=\frac{1}{2}y^{T}K_{y}^{-1}\frac{\partial K_{y}}{\partial \beta_{i}}K_{y}^{-1}y-\frac{1}{2}Tr\left(K_{y}^{-1}\frac{\partial K_{y}}{\partial \beta_{i}}\right)$$

where $\frac{\partial K_{y}}{\partial \beta_{i}}$ is the matrix of elementwise derivatives w.r.t. *β*_*i*_.

The second derivatives are given by

(B2) ∂2∂βi∂βjlogpy|β=12yTKy−1∂2Ky∂βi∂βj−∂Ky∂βjKy−1∂Ky∂βi−∂Ky∂βiKy−1∂Ky∂βjKy−1y−12TrKy−1∂2Ky∂βi∂βj−Ky−1∂Ky∂βjKy−1∂Ky∂βi

and it is the inverse of the negation of the resulting matrix that we use in Eq. (A6).

### C Correction for distortions and movement

When pre-processing the data we used in the present paper we corrected for distortions and movements using a Gaussian process based registration method (Andersson et al., 2012). To use the GP for registration (when data is afflicted by distortions and movement) may seem like boot-strapping, and it is. When data is completely uncorrected the estimate of the error variance hyperparameter (*σ*^2^) will be inflated and the GP fits to the data will be poor. This leads to image predictions that are smooth (both spatially along the PE-direction and in Q-space) compared to the data. Hence, for the first iteration of the correction the observed data will be nudged towards smooth predictions (of themselves). After that iteration there will be initial estimates of distortions and movement that will be used to correct the data prior to the second iteration. Hence when re-estimating the hyperparameters for the second iterations the estimates *σ*^2^ will be smaller and the predictions sharper. That will allow us to further refine the estimates of distortions and movement, *etc*. The full correction procedure progresses like that for a number of iterations (typically five) and at the end of that the estimates for distortions and movement are “finished”, the Gaussian process fits are improved and the image predictions sharper.

The purpose of this paper was not to describe the correction method, but rather how Gaussian processes can be used to model the diffusion signal. Hence we wanted to use corrected example data so as not to mix the two things up.

### D Appendix

The following is the data related to this article.
