# Supplementary figures and images for: Non-parametric representation and prediction of single- and multi-shell diffusion-weighted MRI data using Gaussian processes
Source: Neuroimage. 2015 Nov 15;122:166–76. doi: 10.1016/j.neuroimage.2015.07.067 (PMC4627362; doi:10.1016/j.neuroimage.2015.07.067)

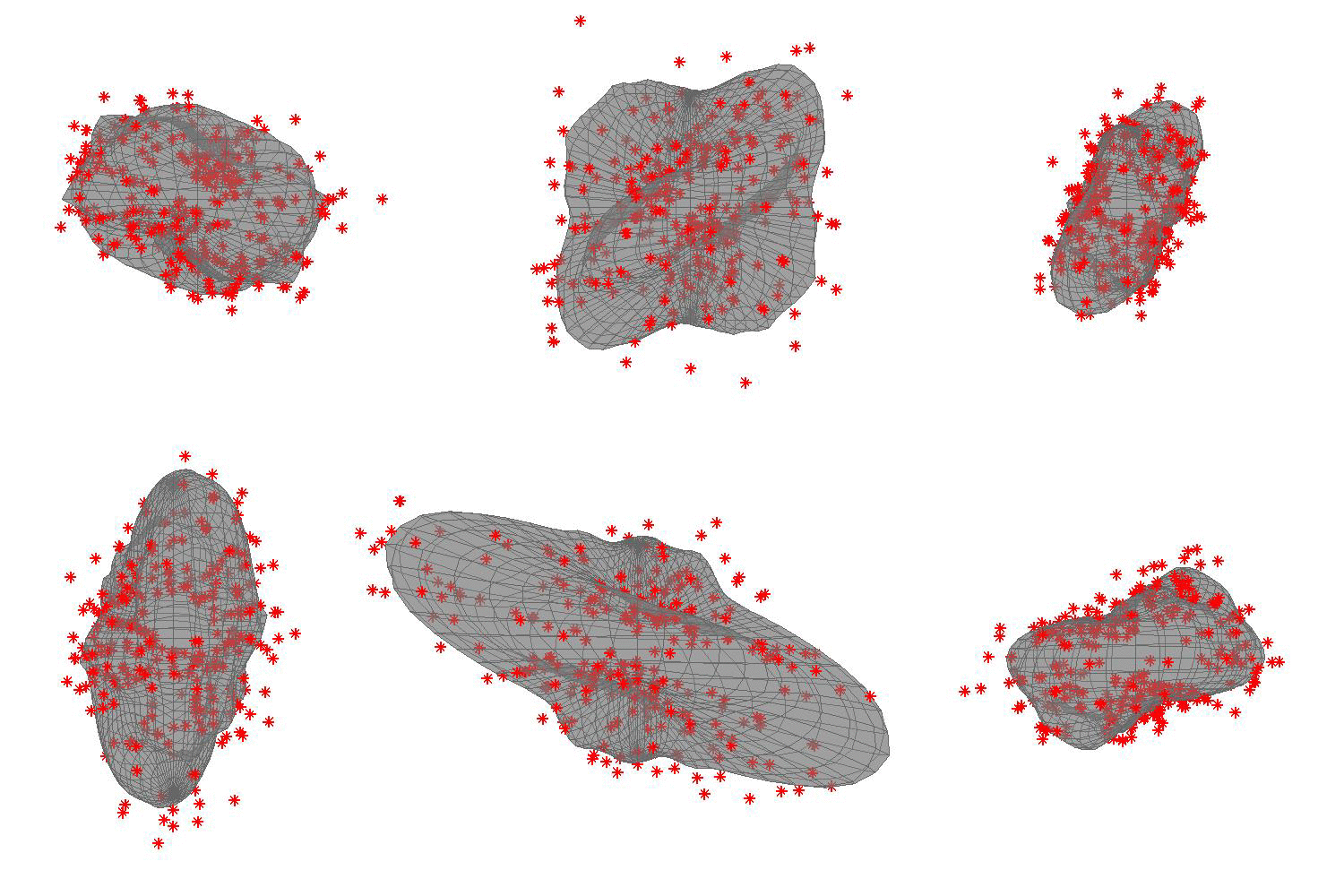

Supplement: Video 1 [file mmc1.gif]
